# The outstanding diagnostic value of DKI in multimodal magnetic resonance imaging for benign and malignant breast tumors: A diagnostic accuracy study

**DOI:** 10.1097/MD.0000000000035337

**Published:** 2023-10-06

**Authors:** Yufei Gao, Yong Wang, Hui Zhang, Xiaolei Li, Lina Han

**Affiliations:** a Department of Radiology, The First Hospital of Hebei Medical University, Shijiazhuang, China; b Department of Radiology, Hebei General Hospital, Shijiazhuang, China; c Hebei Provincial Center for Disease Control and Prevention, Shijiazhuang, China; d Department of Neurology, Hebei General Hospital, Shijiazhuang, China.

**Keywords:** breast tumors, diagnosis, differentiation, diffusion kurtosis imaging, multimodal magnetic resonance imaging

## Abstract

To explore the value of applying different magnetic resonance imaging MRI sequences in the differential diagnosis of benign and malignant breast tumors. Routine breast magnetic resonance scans (T1-weighted image, T1WI; T2-weighted image, T2WI), dynamically enhanced scans, diffusion-weighted Imaging, and diffusion kurtosis imaging (DKI) scans were performed on 63 female patients with breast-occupying lesions. The benign and malignant lesions were confirmed by biopsy, excision-histopathology reports. There are 70 lesions, of which 46 are benign and 24 are malignant. Analyze the primary conditions, such as the shape, size, and boundary of the lesion, and determine the apparent diffusion coefficient (ADC), mean kurtosis (MK), and mean diffusion (MD) values. The receiver operating characteristic curve was used to evaluate the value and difference in differentiating benign and malignant lesions. In this study, the results of the 2 testers both showed that the MK of malignant lesions was significantly higher than that of benign lesions (*P* < .001), and the MD of benign lesions was higher than that of malignant lesions (*P* < .05). The ADC of benign lesions was higher than that of malignant lesions (*P* < .05). For MK, the area under the curve of the 2 testers was 0.855/0.869, respectively. When the best cutoff value of MK for tester 1 was 0.515, the sensitivity and specificity of MK for diagnosing malignant tumors were 83.3%/87.0%, respectively. For the 2 testers MD, and ADC, the area under the curve was < 0.5, and the diagnostic value was low. The MK value obtained by DKI has a specific value in the differential diagnosis of benign and malignant breast lesions. DKI is helpful in the identification of benign and malignant breast tumors. The diagnostic value is outstanding, and its importance to the changes in the microstructure of the organization needs to be further explored.

## 1. Introduction

Breast cancer is one of the most common malignancies in women and has become a significant threat to women’s health.^[1]^ Early detection, early diagnosis, and early treatment are the key to improving breast cancer survival and quality of life.^[2,3]^ In developed countries, breast cancer is the first among female cancers. Although the incidence of breast cancer in China is not as good as that in European and American countries, with the improvement of living standards, the difference between the incidence of breast cancer in China and developed countries is getting smaller and smaller. Different from Chinese breast cancer and European and American countries, Chinese patients are younger, with larger tumor bodies, more positive lymph nodes, and later stage, so early diagnosis is particularly important. Breast lesions examination methods include Mammography, Ultrasonography, CT, and MRI examination, for breast glands with dense breasts, due to the existence of Mammography overlap effect, the lesion occlusion may interfere with the diagnosis, so Mammography in the accurate diagnosis and classification of breast lesions has been facing challenges.^[4,5]^

**Figure 1.**
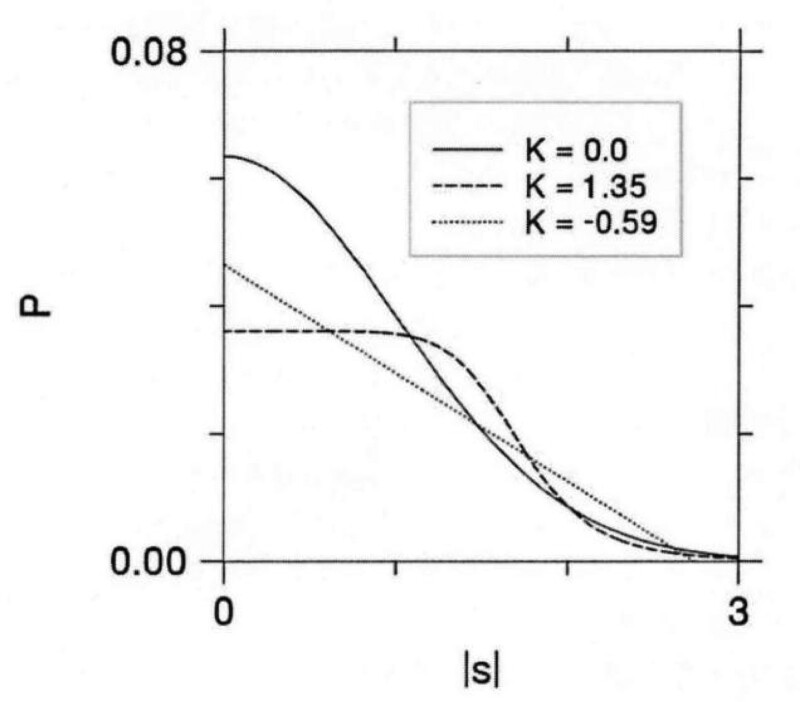
A Gaussian distribution when K = 0; The curve is non-Gaussian when K≠0.

MRI examination benefits to its noninvasive, multi-parameter imaging and high sensitivity to soft tissue, and has obvious advantages for the detection of breast lesions. Dynamic enhanced magnetic resonance technology provides specific morphological and functional information on focal neovascularization through pharmacokinetic modeling techniques, and has been widely used in breast cancer diagnosis and monitoring of tumor response to chemotherapy b. However, the diagnostic specificity of DCE-MRI for breast cancer varies greatly due to background parenchymal enhancement and overlapping time-intensity curves between benign and malignant lesions, which leads to unnecessary biopsies. Furthermore, DCE-MRI may not be suitable for patients allergic to contrast agents or liver and renal insufficiency. In vivo proton MR spectroscopy ([1] H-MRS) is provided with tumor metabolites information to classify tumors, based on the observation of total choline levels. However, total choline has limited sensitivity to distinguishing breast lesion types. The diffusion-weighted Imaging (DWI) single-exponential model and the diffusion tensor imaging model always assume that the water molecules are Gaussian-distributed by diffusion (free and unrestricted diffusion). In fact, at high b values (> 1000 smm2), the complex tissue structure makes the Gaussian no longer fits the Gaussian distribution, and the diffusion signal decay in a non-single-exponential form (Fig. 1). The proposal of diffusion kurtosis imaging (DKI) makes up for the deficiency of DWI and diffusion tensor imaging technology, so early DKI models were used to probe the diffusion behavior of non-Gaussian water molecules in complex tissues, providing valuable information about changes in the tissue microenvironment.^[6]^ The application of DKI has been preliminarily evaluated from glioma grade to lung cancer grade, rectal cancer grade, liver fibrosis, prostate, and spinal benign and malignant lesions identification, and the corresponding research results have been obtained.^[7–10]^ DKI has also been preliminarily applied in the field of breast cancer and combined with different MRI sequences and technologies, and whether DKI technology has more advantages over other sequences is less studied. This study aims to evaluate the differential diagnostic value for benign and malignant breast tumors through the application of different sequences of MR.

## 2. Materials and methods

### 2.1. Clinical data

The prospective study was approved by the Ethics Committee of Hebei Provincial General Hospital. The patients or their authorized relatives provided written informed consent. The case data of patients with breast lesions from October 2019 to September 2020 were collected and sorted out, and 80 patients with DKI sequence scans were screened for analysis. The patient underwent routine MRI examination and DKI scan before surgery; pathological analysis of the lesion was performed postoperatively.

#### 2.1.1. Inclusion criteria

**Figure 2.**
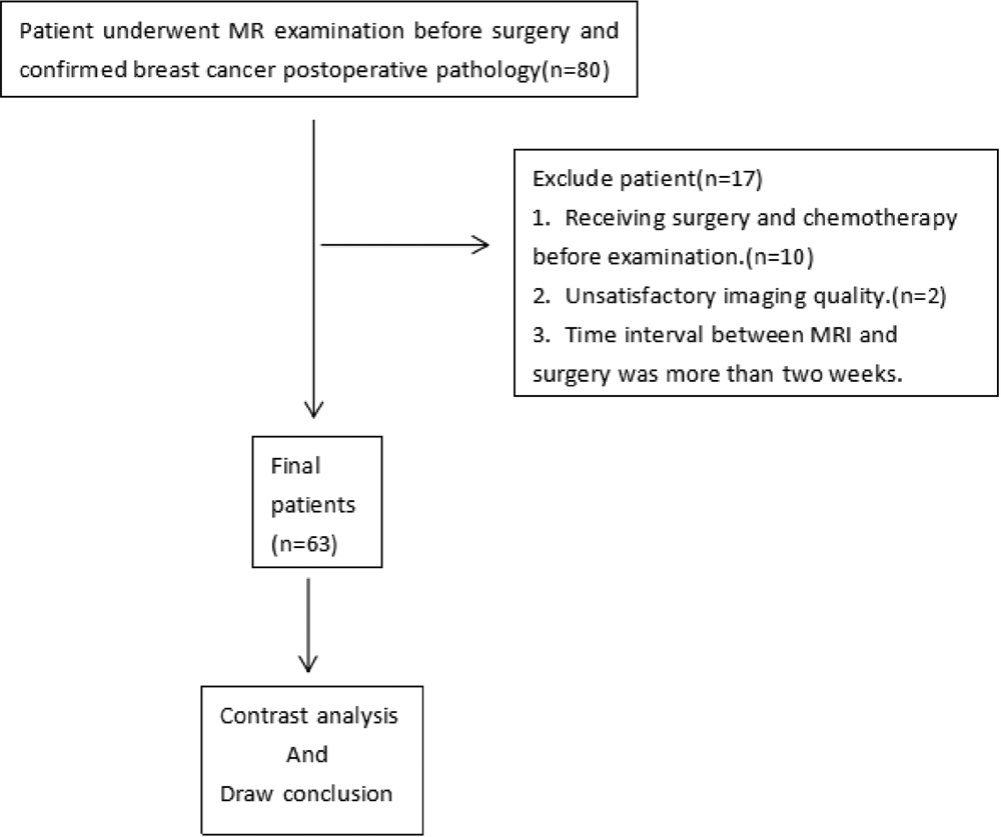
Flowchart of the enrolled patients.

1. No history of release and chemotherapy before examination.
2. No rupture of skin.
3. Precise pathological results obtained within 2 weeks after examination.
4. Clear image obtained by examination and do not affect the diagnosis Finally, 70 lesions in 63 participants were included in the analysis (Fig. 2).

### 2.2. Image acquisition and processing

All MRI examinations were using the 3.0T MRI scanner (Discovery MR750w; GE Healthcare, Waukesha, WI), a 16-channel breast surface phased array coil was used. The patient took the advanced position of the prone head, and the patient remained as motionless as possible and scanned calmly, including all bilateral breast tissue and axillary soft tissue. Premenopausal patients were examined in the second week of the menstrual cycle. An MRI plain sweep and dynamic enhanced scanning were performed in all cases. The contrast was gadolinium spray acid (Gd-DTPA) at a dose of 0.2 m mol/kg at a rate of 2 mL/seconds. For DCE-MRI, 6 phases of T1-weighted images were obtained. The main scan parameters for all sequences are shown in Table 1.

**Table 1:** Scan parameters of each sequence.

Parameter	T2WI	T1WI	DWI	DKI	Dynamic enhancement
TR/TE (ms)	2729/85	697/7.8	2994.3/75.7	5000/91	6.2/1.1
Fat-suppression	STIR	-	SPAIR	SPAIR	SPAIR
Field of view (mm^2^)	36 × 36	36 × 36	36 × 36	24 × 24	36 × 36
Matrix size	512 × 512	512 × 512	256 × 156	512 × 512	512 × 512
Section thickness (mm)	5	5	5	5	1.4
Number of thickness	28	28	28	24	508
B value (s/mm^2^)	-	-	0, 1000	0, 1000, 2000	-
Repetition time	2	1	1,2	1, 2, 3	1

DKI = diffusion kurtosis imaging, DWI = diffusion-weighted imaging, SPAIR = spectral attenuated inversion recovery, STIR = Short TI inversion recovery, TR/TE = repetition time msec/echo time msec.

### 2.3. Measurement and analysis of DWI and DKI data

**Figure 3.**
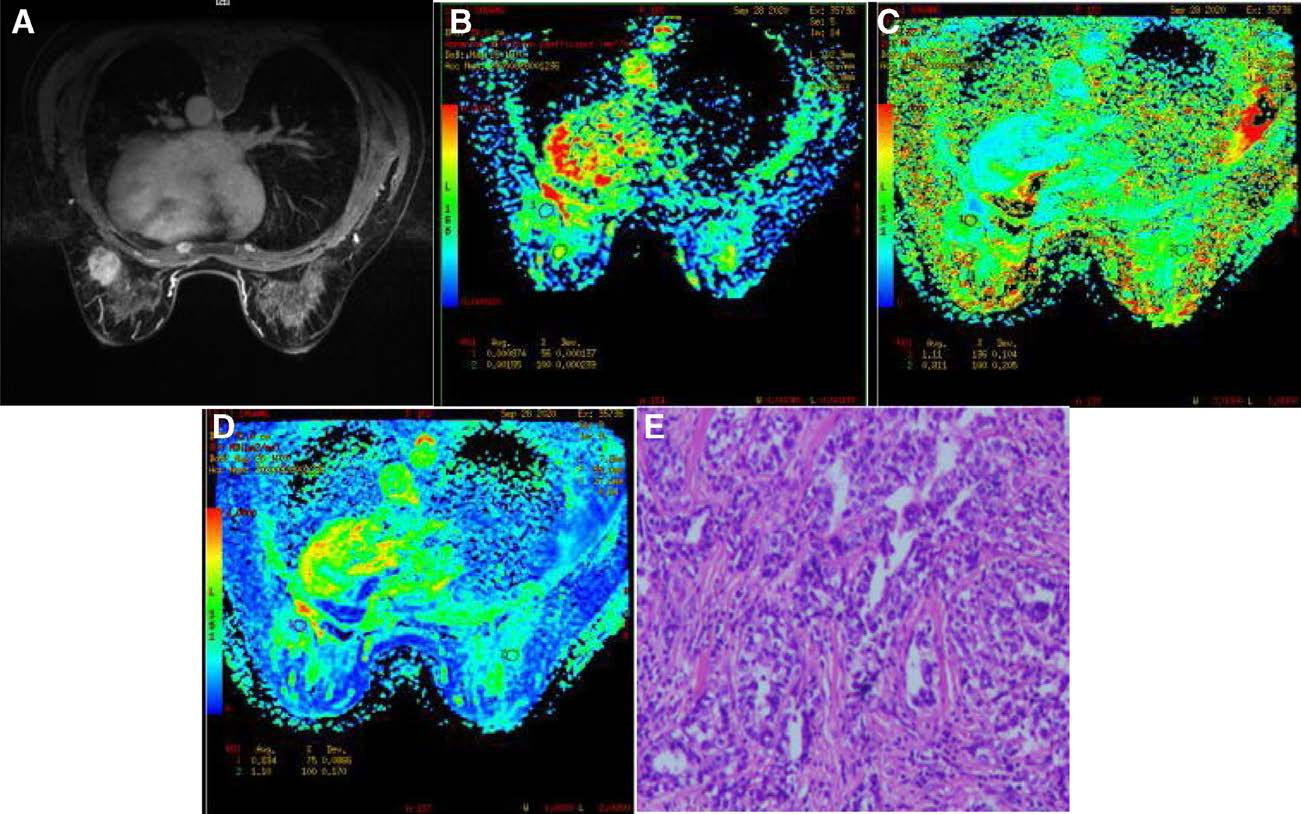
Female, 50-year-old, invasive carcinoma of left breast, nonspecific type, grade WHO III. (A) Axial T1WI enhancement shows the irregular enhancement of the lesion. Pathology of (B) ADC plot, ADC value 0.000874, (C) MK plot, MK value 1.11, (D) MD plot, MD value 0.834, and (E) shows catheter epithelial hyperplasia, disordered cell arrangement and nucleus dysplasia (HE staining, ×200). ADC = apparent diffusion coefficient, MD = mean diffusion, MK = mean kurtosis.

**Figure 4.**
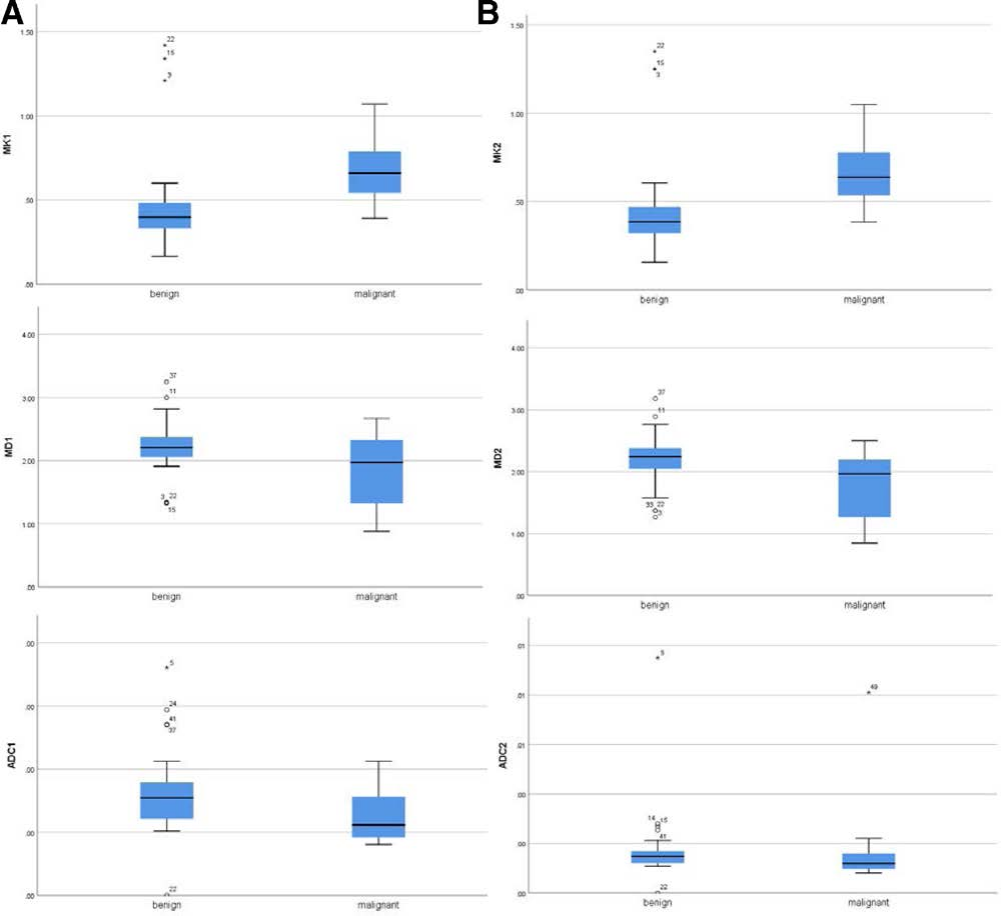
Female, 32, right breast fibroadenoma, (A) axial T1WI enhancement, (B) ADC, ADC = 0.00160, (C) MK, MK = 0.529, (D) MD, MD = 1.85, and (E) microscopic stromal cell hyperplasia, extrusion (HE staining, ×200). ADC = apparent diffusion coefficient, MD = mean diffusion, MK = mean kurtosis.

After all the sequence scanning, the original MRI data is transmitted to the AW4.2 post-processing workstation with Functool software to obtain the apparent dispersion coefficient apparent diffusion coefficient (ADC) diagram and DKI related parameter diagram: mean kurtosis (MK) and mean diffusion (MD) diagram. Two radiologists (tester 1 with 10 years of working experience and reading breast disease; Tester 2 has 5 years of working experience and less experience in breast disease diagnosis) combined with T2WI, DWI, DKI sequence, and dynamic enhancement sequence to delineate the region of interest at the solid component of the lesion region of interest (ROI), Try to avoid liquefied necrosis areas, DWI and DKI parameters for each ROI were measured twice, Taking the average value, The ADC, MD, and MK of the lesion were finally obtained as the final values (Figs. 3 and 4).

### 2.4. Statistical analysis

Data were analyzed using the SPSS 26 software. Metric data that follow a normal distribution are presented as ± seconds. The Mann–Whitney U rank-sum test was used to compare parameters between breast benign and malignant tissues at *P* < .05. The receiver operating characteristic curve (ROC) curve of each parameter was drawn, and the area under the curve (AUC) was calculated to determine the optimal cutoff value of each parameter according to the maximum youden index, and to evaluate the diagnostic efficacy of each parameter on benign and malignant breast lesions.

## 3. Result

The basic condition of the 70 lesions is shown in Table 2. Among them, 46 lesions were benign lesions: adenopathy (n = 13), intraductal papillomas (n = 8), fibroadenomas (n = 15), adenopathy with fibroadenomas (n = 8), fibrous capsular wall-like tissue with massive coagulative necrosis (n = 1), and benign phyllodes tumor (n = 1); There were 24 malignant lesions: invasive ductal carcinoma (n = 15), invasive lobular carcinoma (n = 3), solid papillary carcinoma and crimoid carcinoma (n = 1), invasive crimoform carcinoma with neuroendocrinalization (n = 1), medullary carcinoma (n = 1), liposarcoma (n = 1), mixed ductal lobular carcinoma (n = 1), and apocrine adenocarcinoma (n = 1). General morphological rules of benign lesions; malignant lesions generally have irregular morphology, unclear boundaries, and some lesions can see burr. The benign lesions were mostly inflow enhancement, while the malignant lesions were mostly outflow enhancement.

**Table 2:** Statistical Table of the basic information of the lesions.

Characteristic	Benign group	Malignant group	*P* value
Patient characteristics			
Age^*^	43.6 ± 10.5	52.1 ± 9.9	.002
Menstrual status^*^			.003
Before menopause (n = 57)	42 (73.7%)	15 (26.3%)	
After menopause (n = 13)	4 (30.8%)	9 (69.2%)	
Lesion characteristics			
The mass shape^*^	46	24	*P* < .001
The diameter	15.4 ± 7.7	23.6 ± 10.5	
Class round	40/46 (87%)	2/24 (8%)	
Irregular shape	2/46 (4%)	22/24 (92%)	
Non-mass shape	4/46 (9%)	0	
Internal reinforcement			.117
Well-distributed	7 (15%)	3 (13%)	
Nonuniform	31 (67%)	13 (54%)	
Annular	8 (17%)	8 (33%)	
Dynamic curve type^*^			.014
Inflow type	26 (57%)	7 (29%)	
Outflow type	8 (17%)	15 (63%)	
Flatbed	12 (26%)	2 (8%)	

### 3.1. Analysis of MK, MD, and ADC values of breast lesions

**Figure 5.**
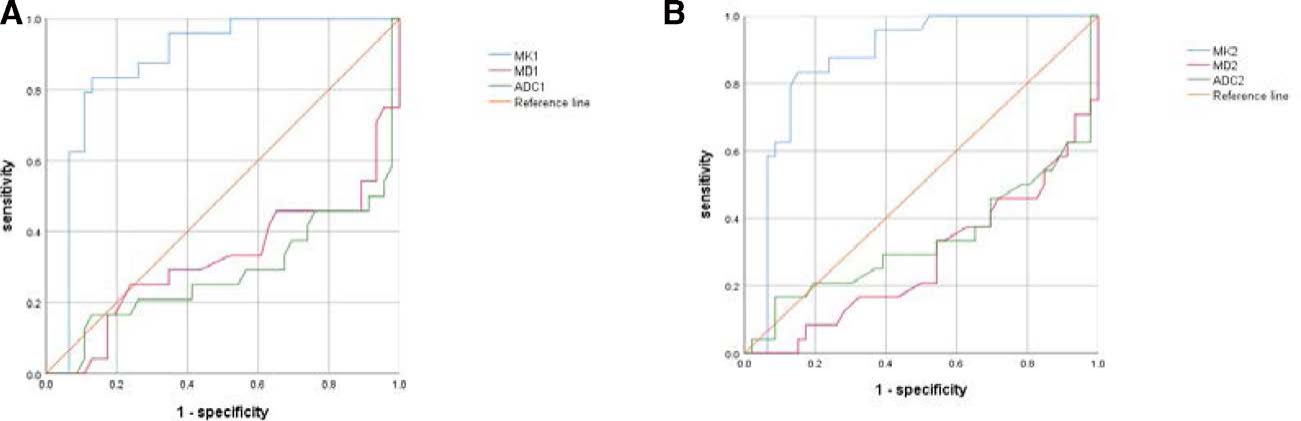
Group a is the resulting boxplot of tester 1; Group b is the resulting boxplot for tester 2.

Statistical analysis of the results measured by different tests showed that: the MK value of benign lesions was lower than malignant lesions; the MD value and the ADC value of benign lesions were higher than malignant lesions, which showed significant differences (*P* < .05, Table 3). Boxplot the test results of the 2 testers respectively according to the test results (Fig. 5). ICC showed the excellent correlation of the 2 subjects, corresponding to MK, MD, and ADC of 0.933, 0.947, and 0.939, respectively.

**Table 3:** Analysis of MK, MD, and ADC values of benign and malignant breast lesions.

	Reader 1			Reader 2		
	Benign	Malignant	*P* value	Benign	Malignant	*P* value
MK	0.46	0.67	<.001	0.46	0.59	<.001
	0.17–1.42	0.38–1.07		0.16–1.35	0.25–1.05	
MD(×10^-3^mm^2^/s)	2.23	1.86	<.001	2.20	1.79	.002
	1.32–3.25	0.88–2.67		1.27–3.18	0.85–2.5	
ADC(×10^-3^mm^2^/s)	1.63	1.27	.007	1.54	1.27	.005
	1.02–3.61	0.81–2.13		1.01–3.65	0.81–2.21	

ADC = apparent diffusion coefficient, MD = mean diffusion, MK = mean kurtosis.

### 3.2. ROC curve analysis

**Figure 6.**
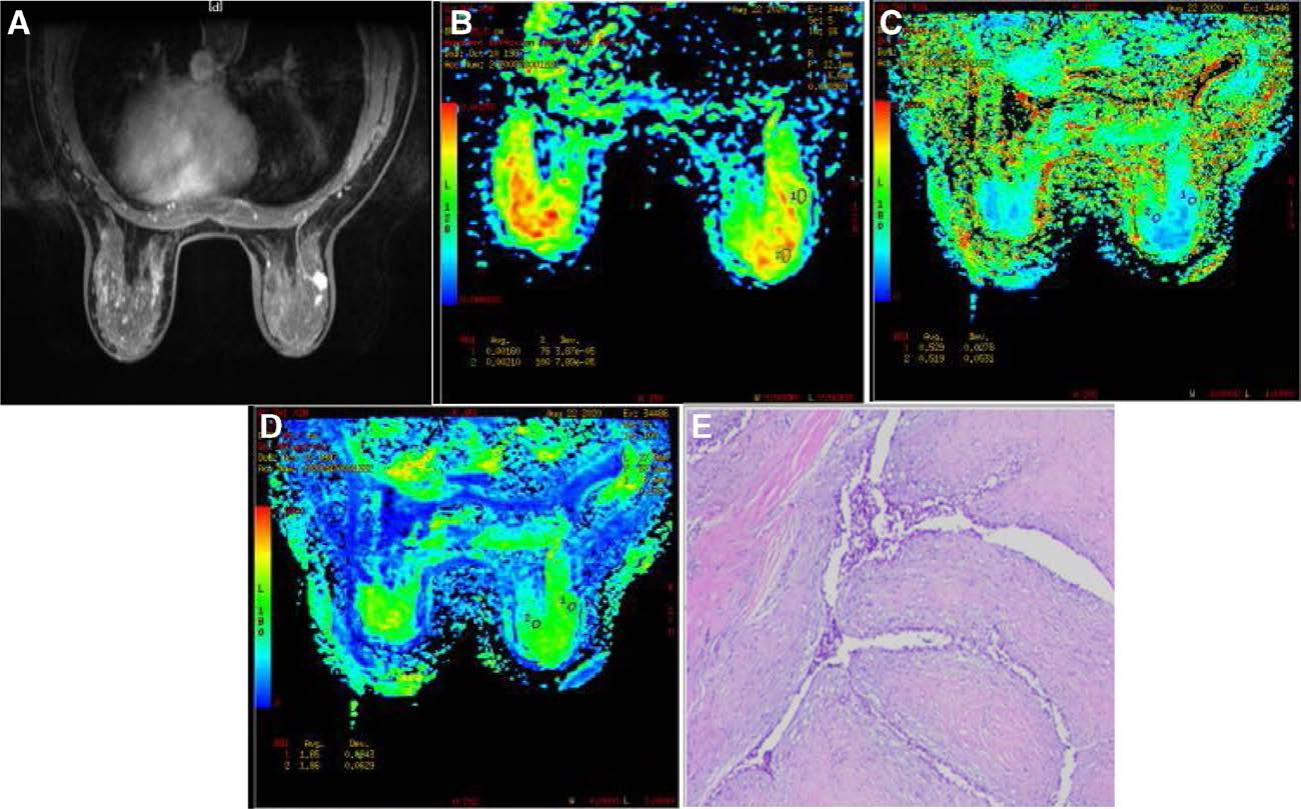
(A) The ROC curve of the tester 1 ADC, MK, and MD values to identify the benign and malignant breast lesions, (B) ROC curve of tester 2 ADC, MK and MD values to identify benign and malignant breast lesions. ADC = apparent diffusion coefficient, MD = mean diffusion, MK = mean kurtosis, ROC = receiver operating characteristic curve.

The ROC curves of the MK, MD, and ADC values were drawn, using the pathological results as the gold standard (Fig. 6). The AUC of MK, MD, and ADC values measured by the 2 testers is 0.855 to 0.869, 0.323 to 0.279, 0.277 to 0.292, respectively; the AUC of MD, and ADC values is < 0.5, which has a low diagnostic value. The sensitivity and specificity of the MK values were 0.833 to 0.833, 0.87 to 0.848, respectively (Table 4). The results show that MK values have the highest sensitivity and specificity and have excellent diagnostic value.

**Table 4:** Diagnostic efficacy analysis of different parameters in distinguishing between benign and malignant lesions.

Parameter	Reader 1				Reader 2			
	AUC	Cutoff	Sensitivity	Specificity	AUC	Cutoff	Sensitivity	Specificity
MK	0.874	>0.515	0.833	0.87	0.869	>0.514	0.833	0.848
MD(×10^-3^mm^2^/s)	0.323	-	-	-	0.279	-	-	-
ADC(×10^-3^mm^2^/s)	0.287	-	-	-	0.336	-	-	-

ADC = apparent diffusion coefficient, AUC = area under the curve, MD = mean diffusion, MK = mean kurtosis.

## 4. Discussion

Our results show that DKI can have outstanding diagnostic value for benign and malignant breast tumors in multimodal MR imaging technology, especially the MK value, which has high sensitivity, specificity, and excellent AUC for benign and malignant diagnosis of breast tumors in quantitative analysis.

DKI can quantify the actual diffuse water molecules and the ideal Gaussian distribution.^[11]^ Indeed, a non-single-exponential diffusion-weighted signal decay with high b values (> 1000 seconds/mm2) has been observed, possibly due to the limited water diffusion associated with the microstructure. Therefore, with the measurement of the diffusion non-Gaussianity of DKI, that is, a measure of diffusion heterogeneity, it can reveal the characteristics of water diffusion characteristics in the tumor microenvironment.^[12,13]^ DKI requires the observation of water molecule diffusion with non-Gaussian distribution behavior at high b values. The DKI model in this study was measured by DWI images with b values of 0,1000 and 2000 seconds/ mm2, involving at least 15 diffusion gradient directions to simulate water molecule diffusion behavior in non-Gaussian models. The change in MD value may be related to the extracellular space volume fraction, reflecting the overall diffusion level and diffusion resistance of water molecules in the tissue, while the diffusion level decreases and the diffusion resistance increases, then the MD decreases. MK can evaluate the extent to which the water molecules with the diffusion displacement distribution deviate from the Gaussian function, reflecting the complexity of the tissue microstructure, the more complex the tissue structure of the selected ROI, the more restricted the water molecules have in their diffusion, and the larger the MK.^[6,14–16]^

According to the results of previous^[10,14,17]^ studies in the literature, The ADC and MD values of the benign lesions were higher than the malignant lesions, and The MK values were higher in malignant lesions than in benign lesions, data from the current study showed that, The ADC value and MD of the benign lesions of the 2 test subjects, which were 1.63 ± 0.5210 to 3 m m2/seconds and 1.54 ± 0.4510 to 3 mm2/seconds, respectively; 2.23 ± 0.37 × 10 to 3 mm2/seconds, 2.21 ± 0.37 × 10 to 3 mm2/seconds, The ADC values and MD values of the malignant lesions were 1.27 ± 0.4510 to 3 mm2/seconds, and 1.27 ± 0.4510 to 3 mm2/seconds, respectively; 1.86 ± 0.54 × 10 to 3 mm2/seconds, 1.79 ± 0.52 × 10 to 3 mm2/seconds, The MK values for benign lesions were 0.46 ± 0.25 and 0.46 ± 0.24, The MK values for the malignant lesions were 0.67 ± 0.19 and 0.59 ± 0.23, Consistent with previous literature reports. In this study, the diagnostic efficacy of MK, MD, and ADC values in malignant breast lesions were all evaluated using the ROC curves. In MK, MD, and ADC values, the results of both tester measurements showed high AUC and specificity of MK values, while the AUC of MD and ADC values were < 0.5, with poor diagnostic efficacy. The ROC curve analysis showed that the MK value AUC was 0.855 to 0.869, with the highest specificity and good diagnostic efficacy, which was consistent with the results of other scholars,^[6]^ it further shows that DKI can more accurately reflect the microstructural changes of the lesions and contribute to the differential diagnosis of benign and malignant breast lesions. The MD and ADC values of benign and malignant breast lesions overlap, while benign lesions, such as ductal papilloma, are closely packed and surrounded by inflammatory reaction, which will limit the diffusion of water molecules,^[18]^ Different from the previous literature, the MD and ADC values in this study did not have a good diagnostic value, and there are several reasons for this situation: In this group, intraductal papilloma and breast adenopathy with partial ductal expansion, secretion retention, lobular and periductal poisoning chronic inflammation MD and ADC values, which may be related to the increased viscosity of extracellular fluid composition caused by rich blood vessels and inflammatory exudation; Higher MD and ADC values in several cases of invasive ductal carcinoma in this group, which may be related to partial hemorrhage, necrosis, cystic change in the central tumor, and lower cell density in the marginal tumor part. Infiltrating ductal carcinoma and ductal carcinoma in situ with or without micro infiltration are the more common types of breast cancer. The higher the tumor grade, the worse the differentiation, and the lower the MD and ADC values. The MD and ADC values also varied between malignancies, including the MD and ADC values that were significantly different in ductal carcinoma or ductal carcinoma in situ with micro infiltration and invasive ductal carcinoma, which was consistent with the study by Kuroki et al^[19]^ MD and ADC values are of certain clinical value in the differential diagnosis of invasive ductal carcinoma and ductal carcinoma in situ.

### 4.1. Insufficiency and outlook

The shortcomings of this study: This study found that the image signal-to-noise ratio decreased under the influence of high b value caused by data noise. Therefore, an advanced sequence to improve SNR is needed; Selected few b values in this study, and there may be some deviation in the analysis results of breast cancer of different pathological types and different levels. In future studies, the number of cases and multiple b values should be expanded in the study, with detailed group analysis of breast cancer according to different pathological types and multiple levels; Breast cancer is a heterogeneous disease, with large differences in cancer cell density and differentiation in different regions, so the selection of ROI may differ from the pathological tissue materials.

## 5. Conclusion

The study results suggest that DKI can provide valuable information on the diffusion properties related to the tumor microenvironment by quantifying the MK values, with more outstanding value than other MRI techniques. DKI can improve the tissue characteristics of breast lesions and increase the diagnostic confidence in breast tumors. Further studies with a larger sample size are necessary to explore the full potential of DKI for noninvasive imaging of breast lesions in a clinical setting. Once well-validated, breast DKI can also be used as a non-contrast breast screening and imaging technology, avoiding unnecessary tissue biopsy, and thus reducing the psychological pressure and economic burden of breast cancer patients to a certain extent.

## Acknowledgements

The authors thank the participants and their families in this study.

## Author contributions

**Conceptualization:** Hui Zhang.

**Data curation:** Yu-Fei Gao, Lina Han.

**Formal analysis:** Yong Wang.

**Resources:** Xiao-Lei Li.

**Software:** Xiao-Lei Li.

**Visualization:** Yong Wang.

**Writing – original draft:** Yu-Fei Gao.

**Writing – review & editing:** Hui Zhang.
